# A novel method to predict essential proteins based on tensor and HITS algorithm

**DOI:** 10.1186/s40246-020-00263-7

**Published:** 2020-04-06

**Authors:** Zhihong Zhang, Yingchun Luo, Sai Hu, Xueyong Li, Lei Wang, Bihai Zhao

**Affiliations:** 1 College of Computer Engineering and Applied Mathematics, Changsha University, Changsha, 410022 China; 2 Department of Ultrasound, Hunan Province Women and Children’s Hospital, Changsha, 410008 China; 3 Hunan Provincial Key Laboratory of Nutrition and Quality Control of Aquatic Animals, Department of Biological and Environmental Engineering, Changsha University, Changsha, 410022 China

## Abstract

**Background:**

Essential proteins are an important part of the cell and closely related to the life activities of the cell. Hitherto, Protein-Protein Interaction (PPI) networks have been adopted by many computational methods to predict essential proteins. Most of the current approaches focus mainly on the topological structure of PPI networks. However, those methods relying solely on the PPI network have low detection accuracy for essential proteins. Therefore, it is necessary to integrate the PPI network with other biological information to identify essential proteins.

**Results:**

In this paper, we proposed a novel random walk method for identifying essential proteins, called HEPT. A three-dimensional tensor is constructed first by combining the PPI network of *Saccharomyces cerevisiae* with multiple biological data such as gene ontology annotations and protein domains. Then, based on the newly constructed tensor, we extended the Hyperlink-Induced Topic Search (HITS) algorithm from a two-dimensional to a three-dimensional tensor model that can be utilized to infer essential proteins. Different from existing state-of-the-art methods, the importance of proteins and the types of interactions will both contribute to the essential protein prediction. To evaluate the performance of our newly proposed HEPT method, proteins are ranked in the descending order based on their ranking scores computed by our method and other competitive methods. After that, a certain number of the ranked proteins are selected as candidates for essential proteins. According to the list of known essential proteins, the number of true essential proteins is used to judge the performance of each method. Experimental results show that our method can achieve better prediction performance in comparison with other nine state-of-the-art methods in identifying essential proteins.

**Conclusions:**

Through analysis and experimental results, it is obvious that HEPT can be used to effectively improve the prediction accuracy of essential proteins by the use of HITS algorithm and the combination of network topology with gene ontology annotations and protein domains, which provides a new insight into multi-data source fusion.

## Background

Proteins play an important role in the life activities of cells. Essential proteins are proteins that can cause cell death or cell infertility if they are missing. Therefore, the identification of essential proteins is important not only for understanding the structure of organisms but also for detection of drug-targets [1] and the prevention of genetic diseases [2]. Methods for identifying essential proteins can be generally divided into two categories. The methods of the first type focus on the use of experimental techniques including single gene knockout [3], RNA interference [4], and genome-wide transposition to mutagenesis of several microorganisms [5]. The drawback of those methods is the expensive price for the biological experiments. The second type of method is computation methods whose costs are far less than the experimental methods. Based on the topological properties of PPI networks, a lot of computational methods such as degree of centrality (DC) [6], information center (IC) [7], closeness centrality (CC) [8], betweenness centrality (BC) [9], subgraph centrality (SC) [10], and neighbor centrality (NC) [11] have been proposed for prediction of essential proteins. The prediction accuracy of these methods is largely influenced by the quality of the PPI network. Unfortunately, most of the PPI networks obtained from high-throughput biological experiments are unreliable and incomplete. In particular, there are a large proportion of PPI networks with false positives. Therefore, some biological data such as sequence data, protein domains, gene expression profiles, protein complexes, and gene ontology (GO) annotations are introduced by researchers to predict essential proteins successively. For example, Hsing et al. [12] developed a method for predicting highly connected central nodes based on GO annotations and interaction data. Ren et al. [13] proposed a prediction model for essential proteins by fusing PPI network topology and protein complex information. Zaki et al. [14] proposed a protein ranking algorithm (ProRank) to quantify the significance of each protein based on the evolutionary relationships and the interaction structure between proteins in the network. Li et al. [15] presented a predictive model of essential proteins based on PPI networks and combining complex centralities. Peng et al. [16] proposed a predictive model, called UDoNC, by integrating protein domain information and PPI networks in yeast. It showed that proteins with more types of self-protein domains tend to be essential. Li et al. and Zhang et al. developed two predictive models called PeC [17] and CoEWC [18], which predicted essential proteins through gene expression and topological characteristics of PPI network. Zhao et al. [19] proposed a predictive model POEM that can measure the essentiality of protein, by detecting overlapping basic modules based on required protein modularity.

The above methods have improved the prediction accuracy by integrating PPI networks and multi-source biological data. They usually constructed a trustable single network by aggregating multiple biological data. However, they ignore the intrinsic correlation between multi-source data. Moreover, different types of interactions may have different effects on the identification of essential proteins. In order to solve this problem, we used the tensor to represent the multi-relationship network [20] of proteins firstly, in which there were multiple interactions between two proteins and each type of interaction has its own unique properties. HITS algorithm was extended from two-dimension matrix to three-dimension tensor model for ranking the score of proteins.

A tensor [21] is a special kind of vector that extends the vector. When the tensor is first-order, it is equivalent to a vector. However, when the order of the tensor becomes higher, it is not equivalent to a high-order vector. The second-order tensor is a matrix, and the third or higher order tensors are collectively referred to as high-order tensors. Obviously, the tensor is well-suited as a model for describing complex networks. Hence, in this paper, according to the concept of tensor, a new method based on tensor and Hyperlink-Induced Topic Search (HITS) algorithm [22] is proposed to predict the essential protein. A tensor model will be established first through fusing GO annotations, protein domains and PPI networks from *Saccharomyces cerevisiae*. Then the HITS algorithm will be extended from a two-dimensional matrix to a tensor model that can be utilized to infer essential proteins. Different from state-of-the-art methods (Pec, CoEWC, and POEM), the importance of proteins and the types of interactions will both do contribution to the essential protein prediction. In addition, the types of protein interactions and the protein scores will affect each other during the iteration process, the protein conservation features derived from orthologous information and the functional features derived from subcellular localization will be considered to generate an initial probability vector as well. Finally, we implement the DIP data [25] to evaluate the predictive performance of our method, and experimental results show that our method is better than other previous central methods such as DC [6], IC [7], CC [8], BC [9], SC [10], NC [11], and three competing methods that integrate network topology features and biological data sources simultaneously such as PeC [17], CoEWC [18], and POEM [23] simultaneously.

## Methods

In this work, we firstly established a tensor model by combining multi-source biological data with a PPI network to reduce negative impacts on prediction. And then, we extend the HITS algorithm from the two-dimensional matrix to the three-dimensional tensor for essential proteins prediction.

### Construction of the protein-protein interaction tensor

**Fig. 1:**
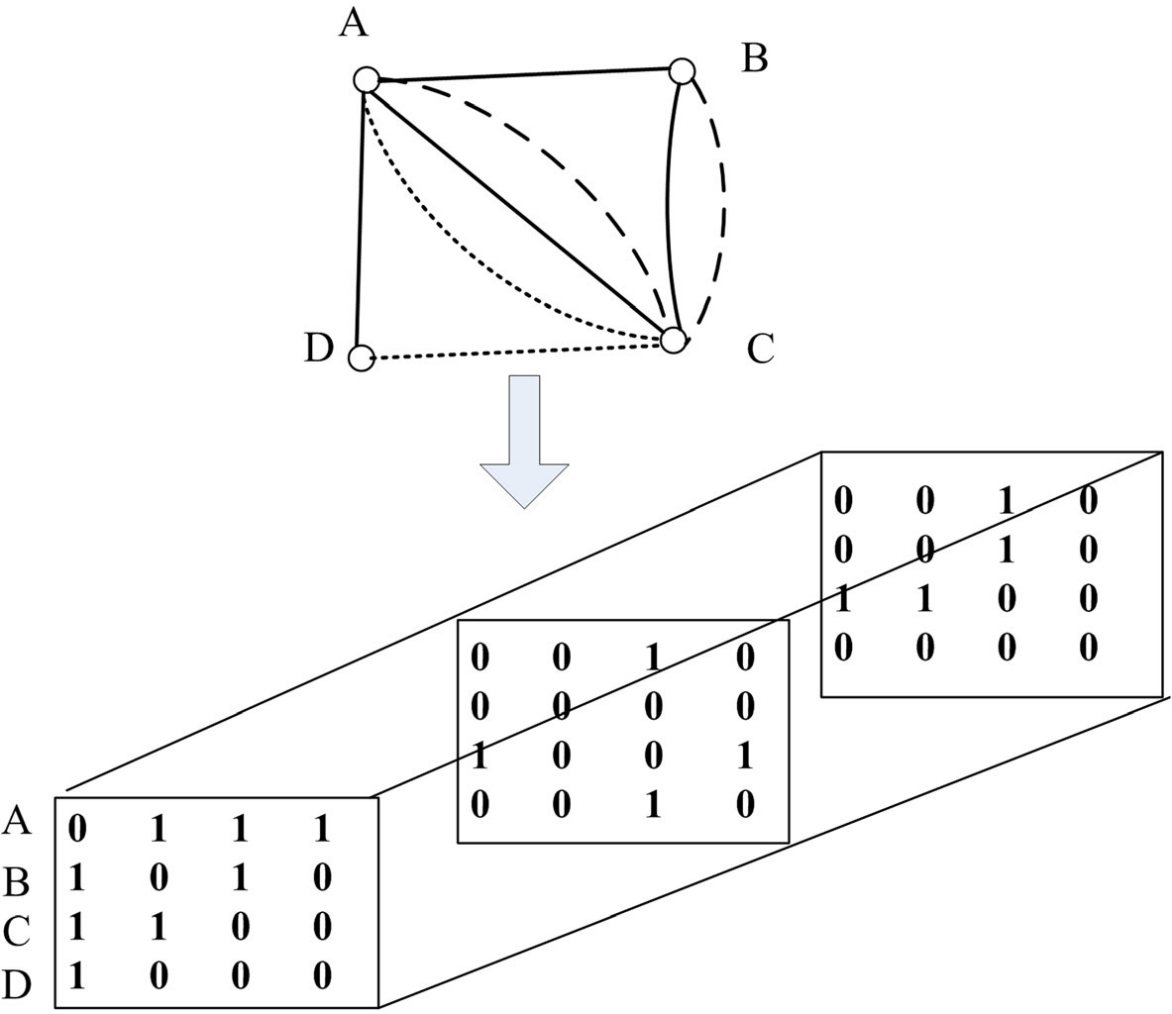
The representation of tensor. It shows a network with four nodes, eight edges and three types of edges. On the bottom side of Fig. 1, each slide represents a kind of connection

An adjacency matrix A can be used to represent a PPI network in which one element represents whether there is an interaction between a pair of proteins. Due to the introduction of multiple biological data sources, there may be more than one interaction between a pair of proteins. Therefore, matrices are not suitable for describing the complex relationships between proteins. Hence, we would adopt tensor to expand the matrix. As shown in Fig. 1, it is obvious that the tensor is more suitable than matrix to represent complex networks with multiple relationships.

We combine protein interaction network topology features, protein domain information, and GO annotations to establish a single-node and multi-relational protein tensor *T* first. Here, the single node is the protein; the multi-relationships refer to the co-neighbor relationships established between the protein pairs based on topological analysis, the co-domain relationships established based on the protein domain information and the co-annotation relationships established based on the GO annotations. The formation process of these relationships will be described in details in the following.

#### 1) The establishment of co-neighbor relationship

The data on protein interactions obtained by high-throughput technology is incomplete. Network topology analysis provides some good ways to overcome these problems. Intuitively, the more co-neighbors between two proteins, the more likely they will interact with each other. In this paper, the proteins *p*_*i*_ and *p*_*j*_ would be considered to be interconnected, if they have at least one common neighbor. This kind of connections between proteins is called the first type of relationships, or the co-neighbor relationships, which can be calculated as follows [23]:

1 $$ Co\_N\ \left({p}_i,{p}_j\right)=\Big\{{\displaystyle \begin{array}{cc}\frac{{\left|{N}_i\cap {N}_j\right|}^2}{\left(|{N}_i|-1\right)\times \left(|{N}_j|-1\right)}\kern0.3em & \mathrm{if}\mid {N}_i\mid >1\kern0.4em \mathrm{and}\mid {N}_j\mid >1\\ {}0&\ \mathrm{otherwise}\end{array}} $$

where *N*_*i*_ and *N*_*j*_ denote the neighborhood sets of *p*_*i*_ and *p*_*j*_ respectively.

#### 2) The establishment of co-domain relationship

Domains may be another clue to the discovery of protein relationships, which it is a stable functional block of proteins, sequences, and structural motifs that exist independently in different proteins. Achieving cellular function requires the cooperation of proteins through many domains. Hence, we can assume that proteins with same domains may interact with the same or similar functions.

Step 1: Calculation of the domain score *P*_*D* of proteins

2 $$ P\_D\left({p}_i\right)=\frac{\sum \limits_{j=1}^{\mid DO\mid}\frac{1}{N{P}_j}\times {t}_{ij}-\underset{1\le k\le \mid P\mid }{\min}\left(\sum \limits_{j=1}^{\mid DO\mid}\frac{1}{N{P}_j}\times {t}_{kj}\right)}{\underset{1\le k\le \mid P\mid }{\max}\left(\sum \limits_{j=1}^{\mid DO\mid}\frac{1}{N{P}_j}\times {t}_{kj}\right)-\underset{1\le j\le \mid P\mid }{\min}\left(\sum \limits_{j=1}^{\mid DO\mid}\frac{1}{N{P}_j}\times {t}_{kj}\right)} $$

where *P* is the set of proteins, DO is the set of different domains in all proteins, and NP_*j*_ is the number of proteins comprising domain *d*_*j*_. If the protein contains the domain *d*_*j*_, then there is *t*_*ij*_ = 1; otherwise, there is *t*_*ij*_ = 0. In addition, in terms of the frequency of the protein domain, the domain score is an important probability of the presence of protein. In the study of this topic, we hypothesized that the basic probabilities of different proteins based on domains are independent of each other.

Step 2: Calculation of co-domain probabilities between pairs of proteins

Based on above assumption, the proteins *p*_*i*_ and *p*_*j*_ would be considered to be interconnected, if they have at least one common domain type. These kinds of connections between proteins are called the second type of relationships, or the co-domain relationships, which can be calculated as follows:

3 $$ Co\_S\left({p}_i,{p}_j\right)=P\_D\left({p}_i\right)\times P\_D\left({p}_j\right) $$

#### 3) The establishment of co-annotation relationship

Considering that proteins participate in functional modules during the molecular processing phase and work with other proteins to perform a function. That is to say, multiple proteins may share functions by participating in the same functional module. Hence, we can use GO annotation to supplement the interaction in the PPI network. For any two proteins *p*_*i*_ and *p*_*j*_ in the PPI network, let *F*_*i*_ and *F*_*j*_ represent the set of functional components of *p*_*i*_ and *p*_*j*_ respectively, and *Co_A (p*_*i*_*, p*_*j*_*)* represents the probability of sharing functions of the two proteins, then it can be obtained as follows [24]:

4 $$ Co\_A\left({p}_i,{p}_j\right)=\Big\{{\displaystyle \begin{array}{ccc}\sqrt{\frac{{\left|{F}_i\cap {F}_j\right|}^2}{\mid {F}_i\mid \times \mid {F}_j\mid }}&, & \mathrm{if}\mid {F}_i\mid >0\ \mathrm{and}\mid {F}_j\mid >0\\ {}0&, & \mathrm{otherwise}\end{array}} $$

Here, *F*_*i*_∩*F*_*j*_ represents a common GO set of proteins *p*_*i*_ and *p*_*j*_.

**Fig 2:**
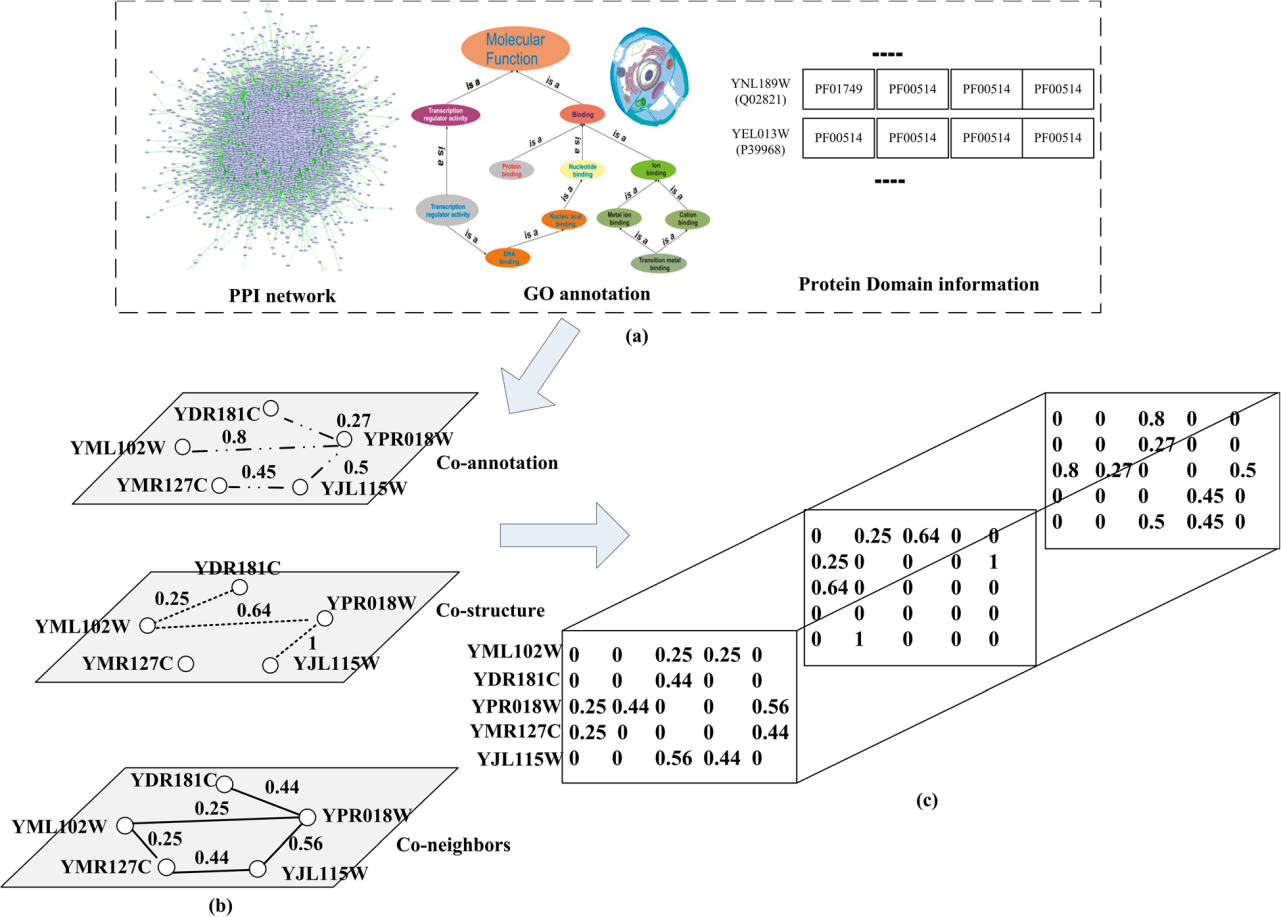
An example of the tensor constructed in this work. An example of the tensor constructed in this work. **a** The input data. **b** The different relationship with three data respectively. **c** The three-relational protein tensor *T*

It is obvious that the tensor *T* can be set correspondingly after forming three connections, as shown in Fig. 2 below.

### Prediction of essential proteins based on the tensor *T*

Based on the fact that some PPI networks have the characteristics of the small worlds, such as the scale-free features and the infinite distance between pairs of nodes, the random walk model is widely used in the prediction of PPI networks. This two-dimensional matrix-based iterative method has also been demonstrated to have excellent performance through experimental results. After building a tensor model to combine the PPI network with multiple data sources, the next key step of our work is to extend the random walk algorithm to multidimensional tensors. Considering that tensor is not only a simple extension of vectors and matrices but also has its own unique properties, so the tensor data processing should be specially processed; otherwise, it will destroy the original data, and the correlation and complementarily are also ignored between different modal data. Thus, a new HITS-based random walk model is proposed to predict the basic protein of protein tensor in this section.

The HITS is a classic random walk algorithm in addition to PageRank. In the HITS algorithm, the importance of a node is measured by an authority value and a hub value, and the two measurements are related to each other. In this paper, the HITS-based random walk algorithm will be extended to the protein tensor model established in the previous stage. Different from two-dimensional matrices, there are many types of associations between proteins in tensors, and each relationship has a different importance for the identification of essential proteins. Based on the principle of HITS algorithm and characteristics of tensor, in our prediction model, we assume the following:

1. If a node is connected by a number of nodes with high hub scores through important edges, it has a higher authoritative score.
2. If a node is connected to many nodes with high authoritative scores through important edges, it has a higher pivotal score.
3. If a type of edge is frequently connected between a high-hub node and a high-authority node, then it has a higher importance.

For convenience, VA, VH, and VE are used to represent the authoritative score vector, the hub score vector, and the importance score vector of different types of edges respectively. Elements in VA and VH are initialized with 1/*n*, while elements in VE are equal to 1/*m*. *n* represents the number of nodes, and *m* represents the number of types of edges between nodes. As described above, there are three different types of edges in our newly constructed interaction network, so *m* = 3.

By performing a normalization operation on the tensor *T*, three probability transfer tensors *T*^*(a)*^, *T*^*(h)*^, and *T*^*(e)*^ can be established, which correspond to the vectors VA, VH, and VE respectively. The calculation formulas are as follows:

5 $$ {t}_{i,j,k}^{(a)}=\Big\{{\displaystyle \begin{array}{c}\frac{t_{i,j,k}}{\sum \limits_{i=1}^n{t}_{i,j,k}}\kern0.75em \mathrm{if}\sum \limits_{i=1}^n{t}_{i,j,k}>0\\ {}1/n\kern2.5em \mathrm{otherwise}\end{array}} $$

6 $$ {t}_{i,j,k}^{(h)}=\Big\{{\displaystyle \begin{array}{c}\frac{t_{i,j,k}}{\sum \limits_{j=1}^n{t}_{i,j,k}}\kern0.75em \mathrm{if}\sum \limits_{j=1}^n{t}_{i,j,k}>0\\ {}1/n\kern2.5em \mathrm{otherwise}\end{array}} $$

7 $$ {t}_{i,j,k}^{(e)}=\Big\{{\displaystyle \begin{array}{c}\frac{t_{i,j,k}}{\sum \limits_{k=1}^m{t}_{i,j,k}}\kern0.75em \mathrm{if}\sum \limits_{k=1}^m{t}_{i,j,k}>0\\ {}1/m\kern2.5em \mathrm{otherwise}\end{array}} $$

In this paper, a novel HEPT method is proposed to predict essential proteins by using the similar power iteration algorithm. For the *t*-th iteration, the three vectors VA, VH, and VE are calculated at step 6-8 shown in algorithm 1.

In above equation (9), *α* is the adjustment parameter and *D* is the jump probability vector, whose value is determined by the homologous score of the protein and the subcellular localization score.

8 $$ D=I(i)\times S(i) $$

Among them, the homology score of protein *p*_*i*_ can be calculated as follows:

9 $$ I(i)=\frac{NI(i)}{\underset{1\le j\le n\mid }{\max}\left( NI(j)\right)} $$

where the molecule is the number of species of the immediate protein containing protein *p*_*i*_, and the denominator is the largest species of all proteins containing the immediate protein.

The subcellular localization formula for protein *p*_*i*_ is as follows:

10 $$ S(i)=\underset{j\in d(i)}{\max}\left(F\_S(j)\right) $$

In the above equation, *d* (*i*) is a subcellular collection of protein *p*_*i*_. *F_S* (*j*) is the score of the *j*-th sub-cell, and the equation is as follows:

11 $$ F\_S(j)=\frac{\mid {p}_j\mid }{\underset{1\le k\le n\mid }{\max}\left(|{p}_k|\right)} $$

where the molecule is the number of proteins containing subcellular *j*, and the denominator is the maximum amount of protein in all subcellular cells.

When the iteration reached a stable state, the proteins were arranged in descending order according to the vector VA. Based on the above description, the overall framework of the HEPT method is as follows in Table 1.

**Table 1:** Overall framework of the HEPT method

Algorithm 1: HEPT method	
**Input:** A PPI network *G*, protein domain, GO annotation, orthologs datasets, subcellular localization datasets; stopping threshold *ε*	
**Output:** Top *N* proteins sorted by *VA* in descending order	
Step 1. Construct the tensor *T* according to Equation (1), (2), (3), (4) Step 2. Calculate jump probability vector *D* with Equation (12), (13), (14), (15)	
Step 3. Construct two transition probability tensors *T*^(*a*)^ , *T*^(*h*)^, and *T*^(*e*)^ with Equation (6)-(8)	
Step 4. Initialize *VA*_*0*_ = 1/*n*, *VH*_0_ = 1/*n*, *VE*_0_ = 1/*m*	
Step 5. Let *t* = 1	
Step 6. Calculate VA_*t*_ = (1 − *α*) × *D* + *α* × *T*^(*a*)^ × VH_*t* − 1_VE_*t* − 1_	
Step 7. Calculate VH_*t*_ = *T*^(*h*)^ × VA_*t*_ × VE_*t* − 1_ Step 8. Calculate VE_*t*_ = *T*^(*e*)^ × VA_*t*_ × VH_*t*_	
Step 9. If ‖VA_*t*_ − VA_*t* − 1_‖ + ‖VH_*t*_ − VH_*t* − 1_‖ + ‖VE_*t*_ − VE_*t* − 1_‖ ≥ *ε*_, then let VA = VA*t*, VH = VH*t*, VE = VE*t*. Otherwise, let *t* = *t* + 1, and then go to Step 6._	
Step 10. Sort proteins by the value of VA in the descending order	
Step 11. Output top *N* of sorted proteins	

In summary, we established a protein interaction tensor by combing PPI networks and multiple biological data. And then, we proposed a new essential proteins prediction method, named HEPT by running the HITS algorithm on the constructed tensor.

## Results and discussion

### I Experimental data

Computational analysis was performed by a PPI network of *Saccharomyces cerevisiae*. Yeast is the most complete and reliable in single cells, and its characteristics have been well demonstrated by knockout experiments. The effectiveness of our proposed method was demonstrated by a detailed introduction to the results of the DIP data set [25]. After self-interaction and repetitive interactions having been filtered out, the DIP data set consisted of 5093 proteins and 24,743 interactions. In addition, there were 1107 different types of domains among the 3042 proteins in the Pfam database [26]. The protein function annotation data was the latest version downloaded from the GO official [27] website. To avoid being too specific or too general, only those GO terms annotated with at least 10 or at most 200 proteins were used for experimental verification, and the number of processed GO terms was 267.

Moreover, the subcellular localization information of proteins used to evaluate proteins was collected from COMPARTMENTS database [28]. The seventh edition of the InParanoid database [29] contained a collection of pairwise comparisons between 100 whole genomes (99 eukaryotes and 1 prokaryote) from which information on orthologous proteins was derived. Additionally, a set of basic proteins used in our experiments were obtained from the MIPS [30], SGD [31], DEG [32], and SGDP [33] databases. Of the 1285 essential proteins, there were 1167 essential proteins in the DIP network.

### II Effect of parameter *α*

**Fig. 3:**
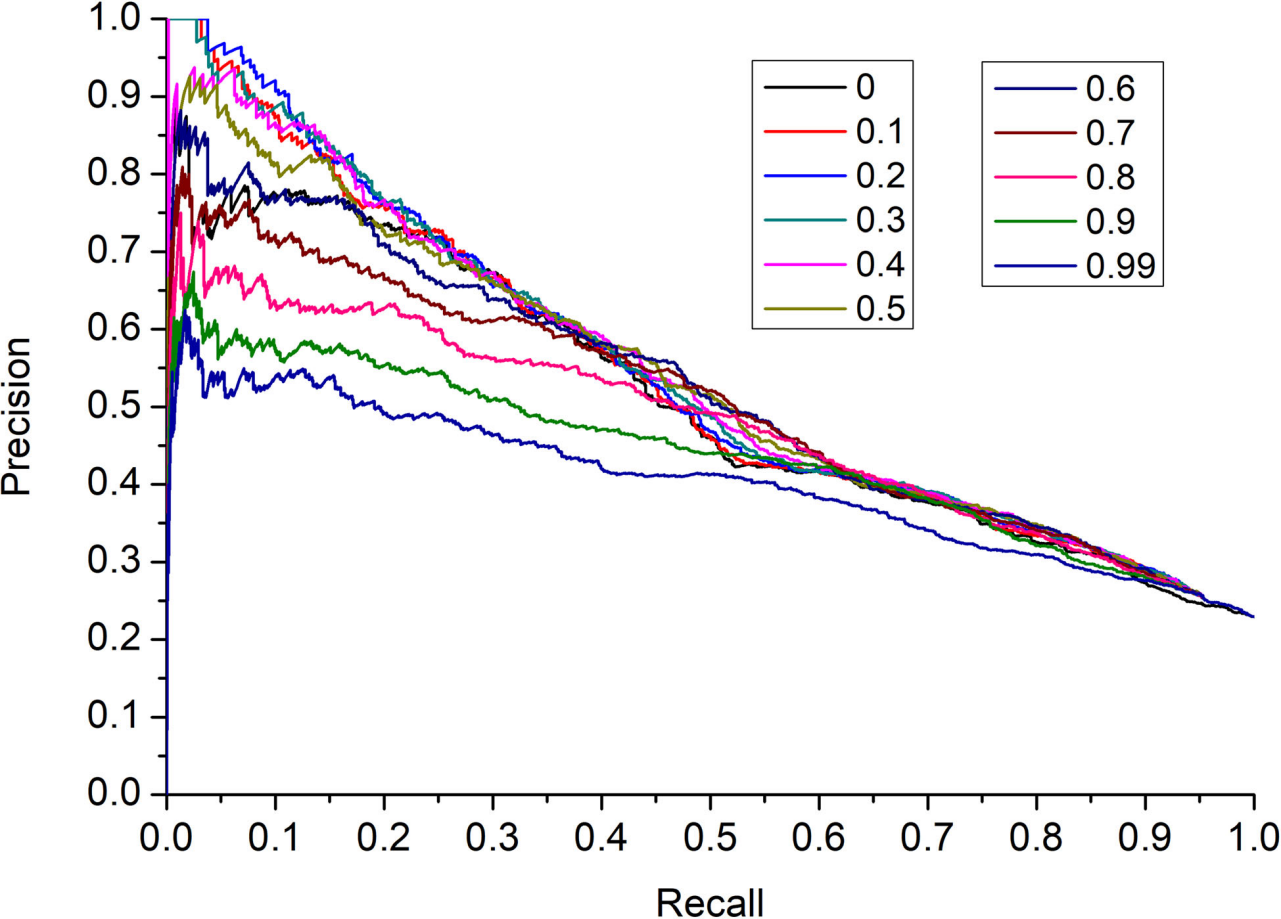
The effect of the parameter α. The figure shows the prediction accuracy of HEPT in each top percentage of ranked proteins by setting different values of *α*, ranging from 0 to 1

In this paper, we introduced the parameter *α* (0 ≤ *α ≤* 1) in Eq. (9). In this section, we adopted a precision-recall (PR) curve to evaluate the effects of the parameter *α* to the performance of our method. And as illustrated in the following (Fig. 3), simulation results showed the comparison results while the parameter *α* was set to different values. Many of the top essential candidates are used to measure prediction accuracy. From observing the following in Fig. 3, it is easy to see that HEPT can archive the highest prediction accuracy when *α* was set to 0.3.

### III Comparison with other methods

**Fig. 4:**
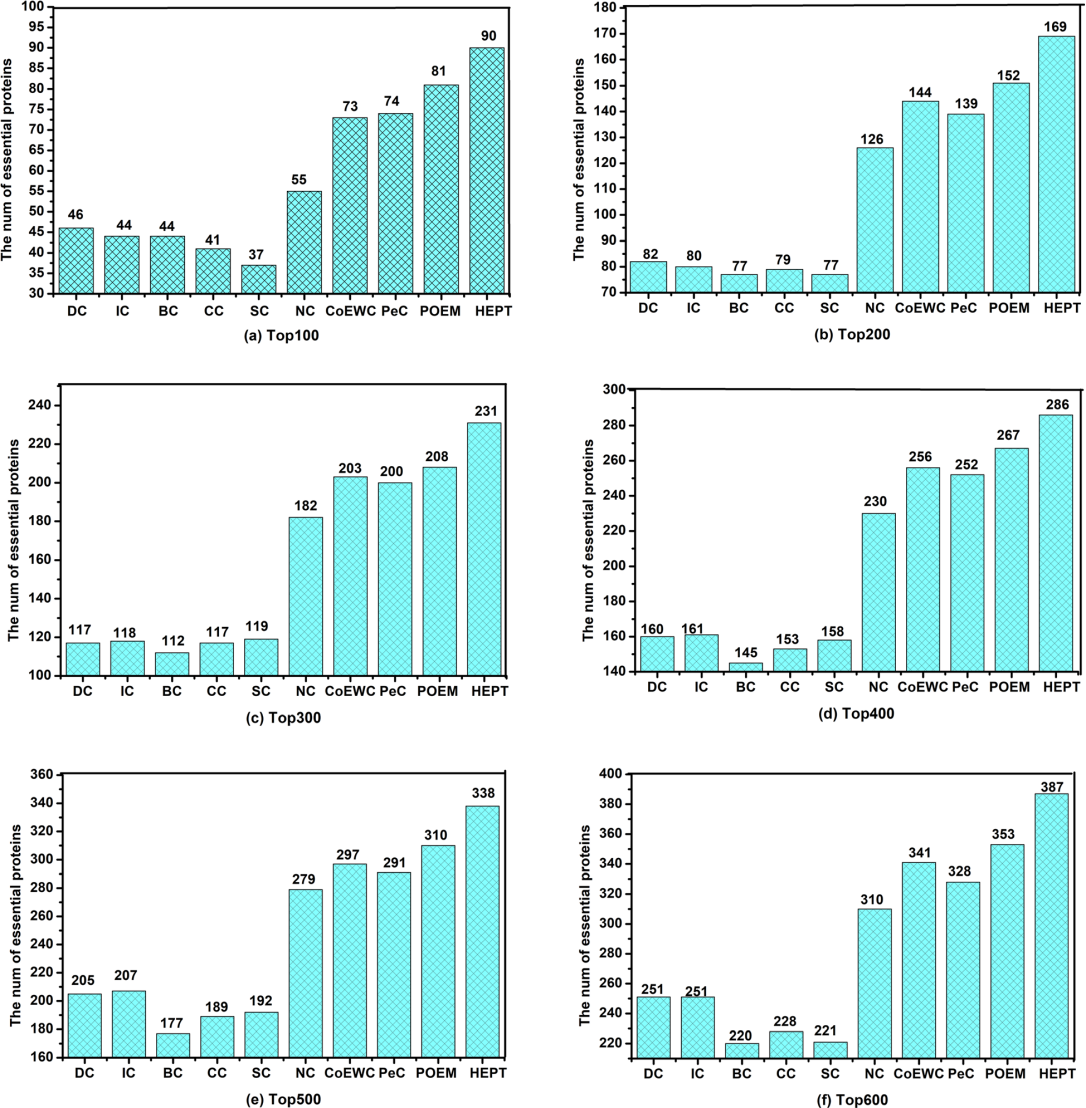
Comparison of the number of essential proteins detected by HEPT and other methods. In order to evaluate the essentiality of proteins in the PPI network, we compared HEPT method with nine existing state-of-the-art central methods such as DC, IC, CC, BC, SC, NC, PeC, CoEWC, and POEM. During simulation, we would perform a score calculation and then sort the scores in descending order. Then, the top ranked scores (including top 100, top 200, top 300, top 400, top 500, top 600) were selected as candidates for verification of essential proteins. **a** Top 100 ranked proteins. **b** Top 200 ranked proteins. **c** Top 300 ranked proteins. **d** Top 400 ranked proteins. **e** Top 500 ranked proteins. **f** Top 600 ranked proteins

We validated the performance of our proposed new method for predicting essential proteins by making a comprehensive comparison of HEPT with a representative set of competitive methods for predicting essential proteins, including DC, IC, BC, CC, SC, NC, PeC, CoEWC, and POEM. The first six methods in the list of competitive methods are classical essential proteins prediction methods, while three other methods discover essential proteins by integrating PPI networks and multiple biological data. The values calculated by the different methods were used to sort the proteins. During simulation, we used a certain number of top proteins as candidates for essential proteins, and then distinguished how many of them were truly essential proteins. The number of essential proteins detected by HEPT and other nine competing methods on the yeast DIP network were shown in the following (Fig. 4).

As shown in Fig. 4, it is obvious that the predictive performance of HEPT is better than all these state-of-the-art competing methods. Among the top100 to top 600 proteins, the predictive performance of HEPT improved 63.64%, 34.13%, 26.92%, 24.35%, 21.15%, and 24.84% respectively, while compared with NC, which had the best performance among the other six topological-based centers such as DC, IC, BC, CC, SC, and NC. In addition, while compared with PeC, CoEWC, and POEM, the predictive performance of HEPT was much better than these state-of-the-art methods as well.

### IV Validated by precision-recall curves

**Fig. 5:**
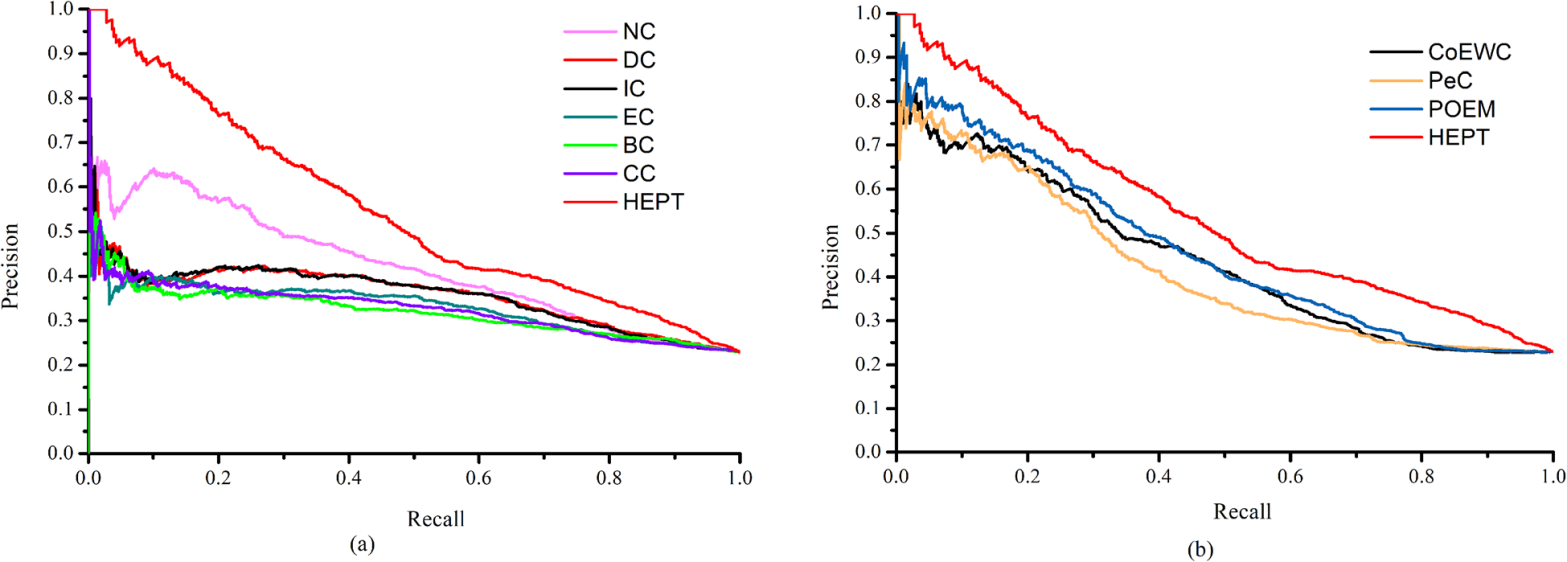
PR curves of HEPT and other existing centrality methods. The scoring of proteins in top *K* (cutoff value) are chosen as candidate essential proteins and the residual proteins in PPI network are seen as candidate nonessential proteins by each method (HEPT, DC, IC, SC, BC, CC, NC, PeC, CoEWC, and POEM). With choosing different values of *K*, the results of precision and recall are calculated for each method respectively. The results of precision and recall are plotted in PR curves with different cutoff values. **a** The PR curves of HEPT, NC, DC, IC, SC, BC, and CC. **b** The PR curves of HEPT and other three methods: CoEWC, PeC, and POEM

In this section, the overall performance of each method was evaluated using a precision-recall (PR) curve. During simulation, the proteins in the PPI network were first ranked in descending order according to the scores calculated by each method. And then the top *K* protein would be selected as the candidate essential protein (positive data set), and the remaining protein was the candidate non-essential protein (negative data set), and the *K* ranged from 1 to 5093. The accuracy and recall values for each method were calculated for different *K* values. Finally, the values of precision and recall values were then in the PR curve with different cutoff values. Figure 5a shows the PR curves for HEPT and six topological-based central methods such as DC, IC, BC, CC, SC, and NC. Figure 5b shows the PR curves including PeC, CoEWC, and POEM for HEPT and other four methods. From observing Fig. 5, it was clear that the PR of HEPT was the best of all methods.

### V Validated by jackknife methodology

**Fig. 6:**
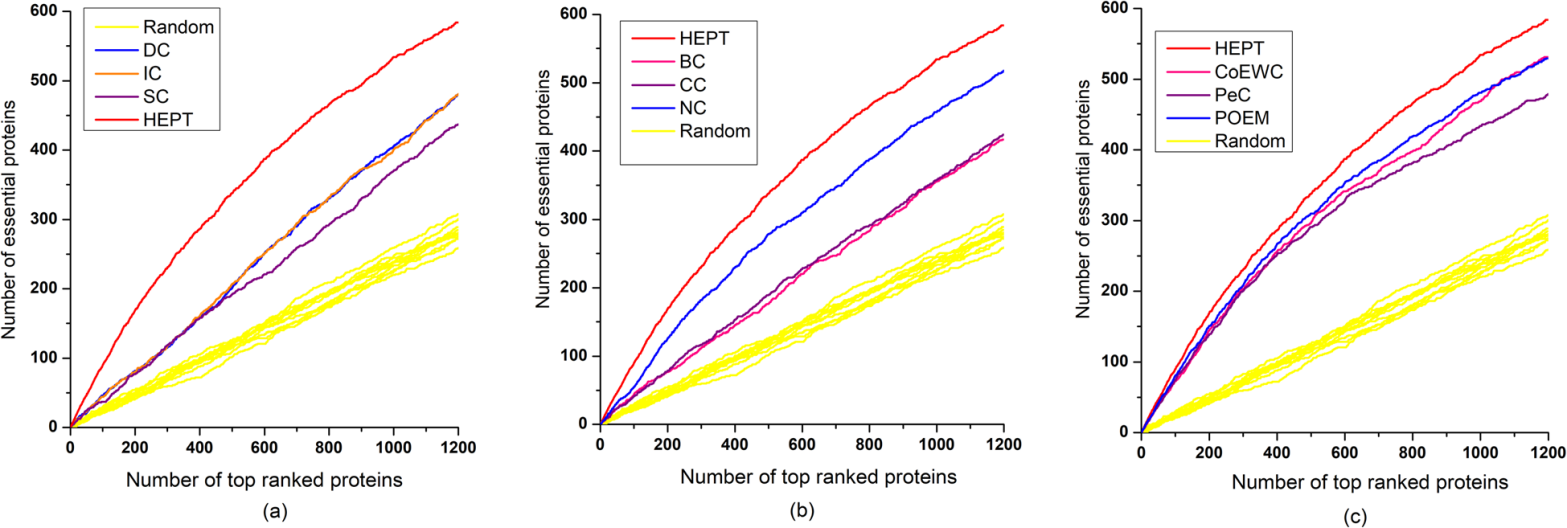
Jackknife curves of ten methods. The prediction performance of HEPT and nine other competing methods (DC, IC, SC, BC, CC, NC, PeC, CoEWC, and POEM) based on protein data are validated by the jackknife methodology. Moreover, the 10 random classifications were drawn for comparison. The *x*-axis represents the proteins in PPI network ranked by HEPT and nine other methods, ranked from left to right as strongest to weakest prediction of essentiality. The *y*-axis is the accumulated count of essential proteins encountered as moving from left to right through the ranked. The areas under the curve for HEPT and the other methods are used for comparing their prediction performance. **a** The comparison results of HEPT, DC, IC, and SC. **b** The comparison results of HEPT, BC, CC, and NC. **c** The comparison results of HEPT and other three methods: PeC, CoEWC, and POEM

In this section, HEPT was further compared to other competing methods (DC, BC, CC, SC, IC, NC, PeC, CoEWC, and POEM) by using jackknife methodology [34]. The area under the folding curve of each method was used to evaluate its prediction performance. In addition, ten random assortments were used for comparison. Figure 6a shows the comparison of HEPT and three central methods (DC, IC, and SC). Figure 6b presents the comparison of HEPT and three topological-based central methods (BC, CC, and NC). Figure 6c illustrates the comparison of HEPT with other three methods (PeC, CoEWC, and POEM). From observing Fig. 6, it is easy to see that the classification curve of HEPT is significantly better than those nine other methods previously proposed. The area under HEPT’s curve is improved 45.80%, 45.76%, 60.15%, 65.87%, 61.78%, 20.63%, 13.64%, 20.25%, and 10.46% than that of DC, BC, CC, SC, IC, NC, PeC, CoEWC, and POEM, respectively. These nine existing state-of-the-art methods also have better predictive performance than random sorting.

### VI Analysis of the differences between HEPT and other methods

In this section, we compared the relationship between HEPT and other competing methods by comparing the top 100 proteins and comparing the prediction accuracy of each protein in different methods to illustrate why and how HPET can achieve good prediction performance. The number of predicted proteins in the top 100 proteins sorted by each pair of methods was given in Table 2.

**Table 2:** Overlap and different proteins predicted by HEPT and other competitive methods ranked in top 100 proteins

Centrality measures (Mi)	|HEPT∩Mi|	|Mi − HEPT|	The non-essential proteins in {Mi − HEPT}	Percentage of non-essential proteins in {Mi − HEPT} with low HEPT value (%)
DC	24	76	50	60.00
IC	26	74	49	61.22
SC	19	81	62	54.84
BC	24	76	50	54.00
CC	25	75	54	57.41
NC	31	69	43	55.81
PeC	43	57	24	87.50
CoEWC	44	56	25	88.00
POEM	45	55	17	82.35

First, we compared HEPT to DC, BC, CC, SC, IC, NC, PeC, CoEWC, and POEM by predicting how many proteins were predicted by HEPT and any of the other nine methods. Table 2 shows the overlap and different proteins of HEPT and one of the other methods. |HEPT∩Mi | was the number of common proteins identified by HEPT and the central method Mi; {Mi-HEPT}, and |Mi-HEPT| were the proteins detected by Mi instead of HPET and the predicted protein quantity respectively.

As shown in Table 2, among the top 100 proteins, common proteins identified by DC, IC, SC, BC, CC, and NC were less than 32%, while the common proteins predicted by HEPT and PeC, CoEWC, and POEM were less than 46%. HEPT and the other nine methods have only a small overlap in the predicted protein, indicating that HEPT is a special method different from other methods. The third column in Table 2 refers to the number of non-essential proteins in different proteins identified by Mi and not identified by HEPT. Further studies of these non-essential proteins predicted by other methods have found that more than 54% of non-essential proteins are lowly rated by HEPT for six central methods based on network topology (DC, IC, SC, BC, CC, and NC), while PeC, CoEWC, and POEM predict that 82% of non-essential proteins also have low POEM scores (less than 0.25).

**Fig. 7:**
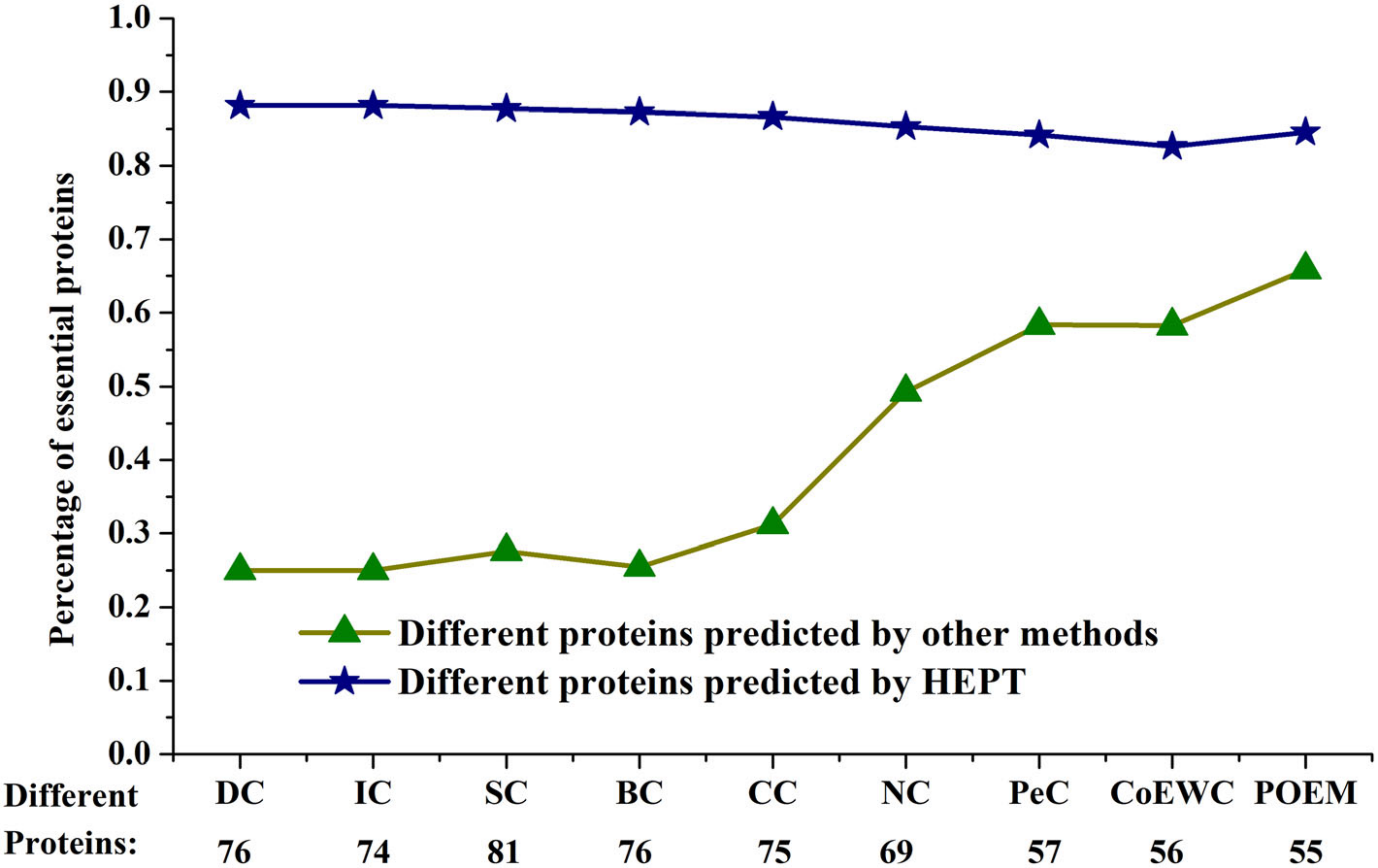
Comparison of the percentage of essential proteins out of all the different proteins between HEPT and other methods. Different proteins between two prediction methods are the proteins predicted by one method while neglected by the other method. The figure shows the percentages of the essential proteins in the different proteins between HEPT and nine other competing methods (DC, IC, SC, BC, CC, NC, PeC, CoEWC, and POEM), respectively

Second, we evaluated HEPT predictions and other methods to predict predictions for different proteins. Figure 7 illustrates the percentage of essential proteins in all different proteins between HEPT and other competing methods. From observing Fig. 7, it was obvious that HEPT performed better than other methods in detecting the percentage of essential proteins. Moreover, SC had the largest number of different proteins from HEPT, and POEM had the smallest difference from HEPT, which were the two most extreme examples. HEPT detected 81 different proteins in all of the top 100 proteins compared to SC, of which 87.73% were essential, while only 27.6% of the proteins detected by SC were essential. In another case, 55 different proteins were identified by HEPT or POEM. HEPT was able to predict that more than 84.55% of the essential proteins were in 22 different protein species, while POEM was less than 65.85%, and the rest of the methods (DC, CC, BC, IC, NC, PeC, and CoEWC) yielded similar results.

## Conclusions

The current calculation methods for detecting essential proteins combined with the network are developed and obtained good performance. But a large proportion of these methods ignored the inherent relationships between multiple organisms meanwhile. In this paper, we filled these gaps by integrating PPI networks, protein domains, and gene expression profiles to construct protein tensors. Moreover, we designed a new random walk model to predict basic proteins by establishing three-dimensional tensors. The experimental results showed that the prediction accuracy of HEPT was better than other competitive methods such as six topological-based central methods and three multi-source data fusion methods. Therefore, to improve the performance of protein prediction through these comparisons, it is necessary to construct a multi-dimensional biological data model and take into account the importance of nodes and different types of edges.

## Data Availability

The datasets used and/or analyzed during the current study are available from the first author or corresponding author on reasonable request.

## Abbreviations

DC: Degree of centrality
IC: Information centrality
CC: Closeness centrality
BC: Betweenness centrality
SC: Subgraph centrality
NC: Neighbor centrality
ProRank: Prtein ranking algorithm
UDoNC: Unite domain and network centrality
PeC: Prediction of essential proteins centrality
CoEWC: Co-expression weighted by clustering coefficient method
POEM: Predictive model based on overlapping essential modules
HITS: Hyperlink-Induced Topic Search
PPI: Protein-protein interaction
PR: Precision-recall
HEPT: A HITS-based method to predict essential proteins from protein tensor
